# Anaphylactoid reactions induced by Shuanghuanglian injection and Shenmai injection and metabolomics analysis

**DOI:** 10.3389/fphar.2023.1200199

**Published:** 2023-07-06

**Authors:** Chi Zhang, Linqi Ouyang, Xili Zhang, Wen Wen, Yuqin Xu, Shan Li, Yingyu Li, Fuyuan He, Wenlong Liu, Hongyu Liu

**Affiliations:** ^1^ The First Hospital of Hunan University of Chinese Medicine, Changsha, China; ^2^ Hunan Key Laboratory of Druggability and Preparation Modification of Traditional Chinese Medicine, Changsha, China; ^3^ College of Pharmacy, Hunan University of Chinese Medicine, Changsha, China

**Keywords:** shuanghuanglian injection, shenmai injection, anaphylactoid reactions, LC-MS metabolomics, metabolic pathways

## Abstract

**Introduction:** Shuanghuanglian injection (lyophilized) (SHLI) is commonly used to treat respiratory tract infection. Shenmai injection (SMI) is mainly used to treat cardiovascular diseases. Despite their widespread clinical use, anaphylactoid reactions (ARs) induced by SHLI and SMI have been reported, which have attracted broad attention. However, the impact of ARs on metabolic changes and the underlying mechanisms are still unclear.

**Methods:** ICR mice were used as model animals and were treated with normal saline, C48/80, SHLI and SMI, respectively. The behavior of mice, auricle blue staining and Evans Blue exudation were used as indexes to evaluate the sensitization of SHLI and SMI and determine the optimal sensitization dose. Anaphylactoid mice model was established based on the optimal dose and enzyme-linked immunosorbent assay (ELISA) was used to model verification. Afterwards, plasma samples of administered mice were profiled by LC-MS metabolomics and analyzed to evaluate the changes in metabolites.

**Results:** High doses of both SHLI and SMI can induce severe anaphylactoid reactions while the reaction induced by SMI was weaker. A Partial Least-Squares Discriminant Analysis (PLS-DA) score plot indicated that following administration, significant metabolic changes occurred in mice. 23 distinct metabolites, including deoxycholic acid, histamine, and 5-hydroxytryptophan, were identified in the SHLI groups. 11 distinct metabolites, including androsterone, 17α-hydroxypregnenolone, and 5-hydroxyindoleacetate, were identified in the SMI groups. Meanwhile, different metabolic pathways of SHLI and SMI were predicted by different metabolites. The associated metabolic pathways include steroid hormone biosynthesis, tryptophan metabolism, histidine metabolism, arachidonic acid metabolism, nicotinate and nicotinamide metabolism, and primary bile acid biosynthesis.

**Conclusion:** Study showed that both SHLI and SMI can induce varying degrees of anaphylactoid reactions, a positive correlation between response intensity and dose was observed. Metabolomics showed that SHLI and SMI may promote the simultaneous release of hormones and inflammatory factors by disturbing relevant metabolic pathways, while SMI may also inhibit the release of inflammatory factors in arachidonic acid metabolic pathway, indicating both pro-inflammatory and anti-inflammatory effects. This study will serve as a reference for developing a new approach to evaluate the safety of SHLI and SMI from perspective of susceptible drug varieties. However, ARs mechanism requires further verification.

## 1 Introduction

**FIGURE 1:**
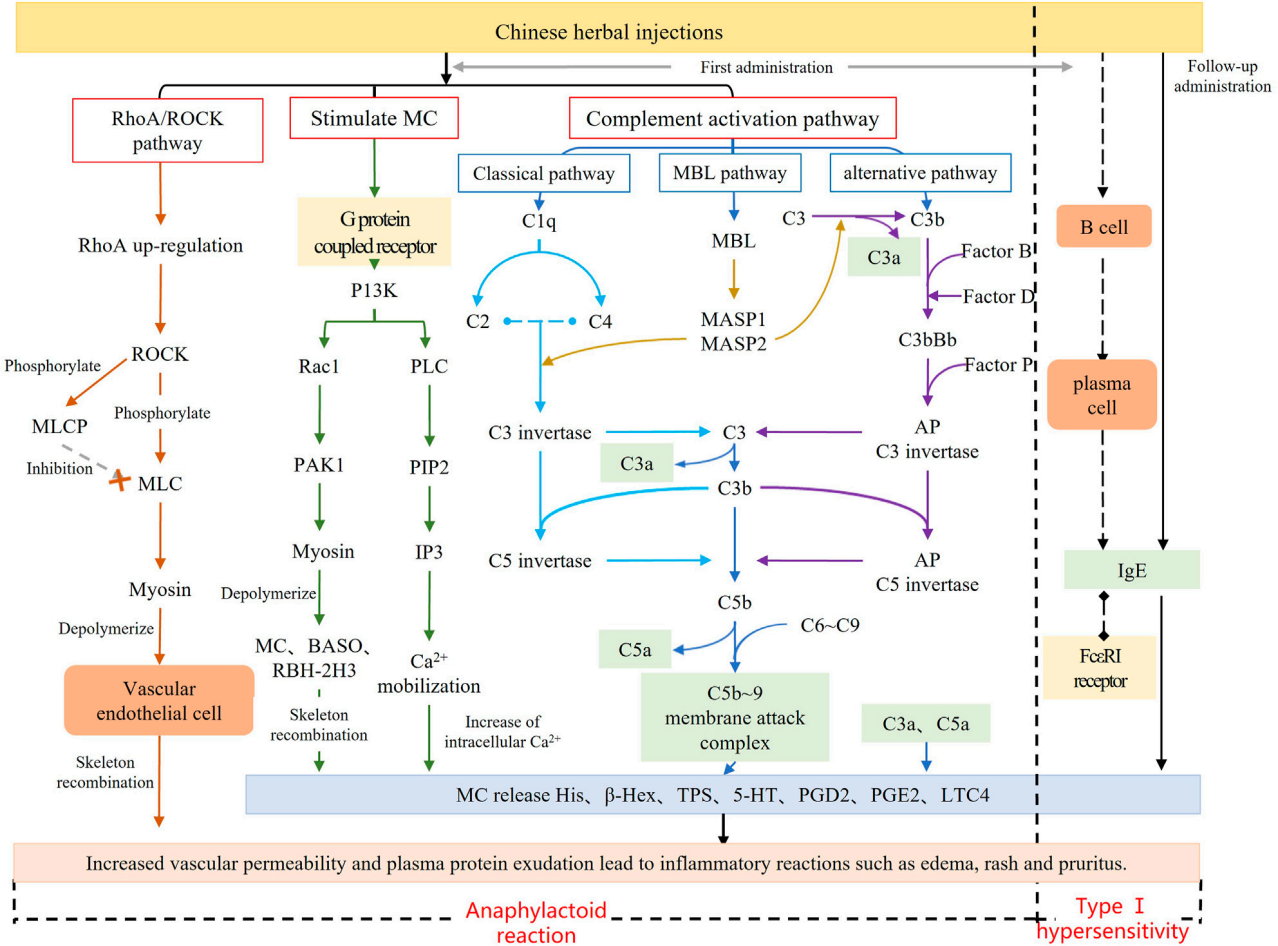
Mechanism diagram of anaphylactoid reaction.

Traditional Chinese medicine injection (TCMI) has the advantages of high bioavailability and rapid action, which broaden the range of conventional drug delivery methods and accelerate the growth of TCMI. (Li and Zhang, 2011). However, there has been an annual increase in the number of clinical reports of adverse reactions (ADRs) caused by TCMI, (Zhang et al., 2014; Sun et al., 2013; Huang R. et al., 2021), with anaphylactoid reactions (ARs) accounting for more than 77% (Xu and Dou, 2015). Different from type Ⅰ hypersensitivity, ARs occur rapidly after the first exposure to the antigen and are not initiated or mediated by pre-existing IgE (Yarema et al., 2018; Li et al., 2020) (Figure 1). The clinical symptoms of ARs resemble those of systemic anaphylaxis, typically presenting as pruritus, rash, papules, shortness of breath and asthma, etc. (Farnam et al., 2012; Yarema et al., 2018). Currently, few studies available on injectable-induced ARs. Previous studies have predominantly concentrated on *in-vitro* studies on animal-related behavioral symptoms and sensitizing components including injectable additives and major active ingredients (Xiang et al., 2013; Wang et al., 2017; Xu et al., 2017). The active ingredients of TCMI are highly complex. In addition to exogenous components such as active ingredients or injection additives, metabolites of medicine components may also act as allergens for ARs. Consequently, ARs of TCMIs may be triggered by multiple pathways and targets, with distinct characteristics and mechanisms observed among different injections. As a systematic approach, metabolomics has become an important tool for elucidating the mechanisms of ARs (Xu et al., 2015; 2020; Chen et al., 2022). To enhance the study of ARs induced by TCMI, it is necessary to use metabolomics to identify potential biomarkers and systematically reveal the mechanism.

Shuanghuanglian injection (SHLI) is a classical TCMI composed of *Scutellaria baicalensis* Georgi [Lamiaceae; Scutellariae radix], *Forsythia suspensa* (Thunb.) Vahl [Oleaceae; Forsythiae fructus] and *Lonicera japonica* Thunb [Caprifoliaceae; Lonicerae japonicae flos]*.* It mainly contains the active ingredients of chlorogenic acid, baicalin and forsythia glycosides (Ma et al., 2017; Huang P. et al., 2021). Over the years, it has been proven to be effective in treating respiratory diseases caused by bacteria or viruses (Zhang et al., 2013; Tang et al., 2018; Su et al., 2020). Shenmai injection (SMI) is another TCMI prepared from the extracts of *Panax ginseng* C.A.Mey [Araliaceae; Ginseng radix et rhizome rubra] and *Ophiopogon japonicus* (Thunb.) Ker Gawl [Asparagaceae, Ophiopogonis radix], with ginsenosides as the main active ingredients (Olaleye et al., 2019). SMI is extensively used in the clinical treatment of coronary heart disease, viral myocarditis, heart failure, respiratory failure, and cerebral infarction (Yao et al., 2017; Yuan et al., 2019). In recent years, there has been an increase in the number of ADR reports for SHLI and SMI (Wang et al., 2010). This has, to some degree, limited their clinical application.

In this study, we selected SHLI (lyophilized) and SMI as model drugs, and hyper-aphylactic ICR mice as model animals to investigate the distinct characteristics and explain the differences in SHLI and SMI-induced ARs using LC-MS metabolomics. We aim to identify endogenous potential biomarkers associated with ARs caused by SHLI and SMI, and finally elucidate ARs mechanism in biological metabolic level. This study will serve as a reference for developing a new approach to evaluate the safety of TCMI from perspective of susceptible drug varieties.

## 2 Materials and methods

### 2.1 Animals

SPF male ICR mice (25–30 g, Slake Jingda experimental animal Co., Ltd., Hunan, China) were maintained under the standard animal room conditions (temperature 20–26°C; humidity 40–70% and 12 h light-dark cycle) in the barrier environment of the Experimental Animal Center of Hunan University of Traditional Chinese medicine (Changsha, China).

### 2.2 Experiment and ethics statement

All procedures performed on animals were approved by the Institutional Animal Ethics Committee (License number: SYXK 2019–0009; Ethics Review number: 201910090002). The use of animals was compliant with the National Research Council’s Guide for the Care and Use of Laboratory Animals published by the US National Institutes of Health (NIH Publication No. 85-23, revised 1996). The research was compliant with The Convention on Biological Diversity and the Nagoya Protocol.

### 2.3 Reagents and materials

Shuanghuanglian for injection (lyophilized) (600 mg) was purchased from Harbin Pharmaceutical Group No. Two Traditional Chinese medicine factory (Harbin, China) (Batch number: 1904002). Shenmai injection (10 mL) was purchased from Chiatai Qingchunbao Pharmaceutical Co., Ltd. (Hangzhou, China) (Batch number: 1912094). Compound 48/80 (C48/80) was purchased from Sigma Aldrich (Saint Louis, MO, USA) (Batch number: 088M4120V). Mouse Histamine ELISA Kit was purchased from Elabscience Biotechnology (Wuhan, China) (Batch number: DPM78G9DWG). Mouse tryptase Kit was purchased from Jianglai biology Co., Ltd. (Shanghai, China) (Batch number: April 2020). Chromatographic pure methanol, formic acid, ammonium acetate was purchased from Thermo Fisher Scientific (Waltham, MA, United States). Normal saline (NS) for injection was purchased from Hunan Kelun Pharmaceutical Co., Ltd. (Yueyang, Hunan, China) (Batch number: 088M4120V). Evans Blue (EB) was purchased from Shanghai Macklin Biochemical Co., Ltd. (Shanghai, China).

### 2.4 Drug injection preparation

Normal saline was used as solvent to prepare two concentration gradients of C48/80 injection (2.3 mg/mL and 2.5 mg/mL) and three concentration gradients of SHLI (30 mg/mL, 60 mg/mL and 120 mg/mL). NS, C48/80, SHLI and SMI were prepared into injection containing 0.4%EB.

### 2.5 Exploration of anaphylactoid dose of SHLI and SMI

Based on the standard therapeutic dose of drugs for humans (SHLI: 60 mg kg^-1^; SMI: 20–100 mL d^-1^), the injection doses of mice were then obtained according to the conversion formula of body surface area between mice and humans. We set low, medium, and high doses of SHLI at 0.5x, 1x, and 2x the standard dose. For SMI, we set low, medium, and high doses at the lowest, intermediate, and maximum standard doses. Hyper-aphylactic ICR mice were randomly divided into five groups, with 8 mice in each group, and sensitized through a single tail vein infusion. Mice in Group 1 (NS-1, blank control) were injected with NS (10.8 mL/kg). Positive control group (C48/80–1) were injected with C48/80 (10.8 mL/kg). Mice in groups 3–5 represented animals receiving experimental drug, the groups are the SHLI 0.5x standard dose group (323 mg/kg, SHL-1), 1x standard dose group (647 mg/kg, SHL-2), and 2x standard dose group (1,293 mg/kg, SHL-3), respectively. The other 40 mice were also divided into five groups: a blank control group (NS-2, 10 mL/kg), a positive control group (C48/80–2, 10 mL/kg), low, medium, and high dose groups of SMI (SM-1, 3.6 mL/kg; SM-2, 10.8 mL/kg; SM-3, 18.0 mL/kg).

The behavioral changes of mice were observed and recorded within 15 min after administration. These changes were then analyzed in combination with the classification criteria of anaphylactoid reaction symptoms (Table 1) and references (Han et al., 2015; Zhang et al., 2015; Dai et al., 2017). Following the behavioral observations, mice in each group were sacrificed, and their ears were collected. The response rates of auricle blue staining in each group were calculated (the response rate of auricle blue staining = the number of animals with auricle blue staining/the number of animals in the group ×100%). Additionally, the blue-stained area (S) of auricle was scored according to the standard outlined in Table 2 (Liang et al., 2012). After scoring, cut the auricle into pieces, add 2 mL of mixture of acetone and NS, and soak it in the refrigerator at −20°C overnight. Dilute the mixed solution of EB mother liquor and acetone:NS mixture into solutions with concentrations of 0.016, 0.008, 0.0032, 0.0016, 0.0008 and 0.0004 mg/mL, respectively. Measure the absorbance at 610 nm using a UV spectrophotometer and establish the standard curve EB UV absorption. Centrifuge the preserved mouse binaural extract at 3000 rpm for 15 min. Then take the supernatant and measure the absorbance at 610 nm. Finally, calculate the EB exudation of each group using the standard curve.

**TABLE 1:** Classification criteria of anaphylactoid reaction symptoms.

Reaction symptoms	Score	Level
normal	0	negative
Restlessness, reduced spontaneous activity, bristling, shaking and scratching the nose	1	weakly positive
Sneezing, coughing, shortness of breath, urination, defecation, tears	2	positive
Dyspnea, wheezing, purpura, unstable gait, jumping, wheezing, spasm, rotation	3	strong positive
death	4	extremely strong positive

**TABLE 2:** Scoring standard of mouse auricle blue staining.

S	Score
0(No blue dye)	0
0 < S ≤ 1/8	1
1/8 < S ≤ 1/4	2
1/4 < S ≤ 1/2	3
1/2 < S ≤ 3/4	4
3/4 < S ≤ 1	5

The software SPSS 26 was used for statistical analysis of behavioral response ratings, auricle blue staining rates and EB exudation of mice. One way ANOVA was used to test the difference between groups that followed a normal distribution, and Kruskal Wallis was used to test the difference between groups if it did not obey the normal distribution. The experimental results were expressed in *P*, and *p*-values <0.05 were considered statistically significant.

### 2.6 Establish ARs model and verification

SHLI and SMI test solutions were prepared in optimal ARs dose obtained from the previous section. Except for SMI, drugs have been prepared with normal saline to the concentration of the same administration volume (i.e. 10 mL/kg). Mice were randomly divided into four groups: blank control group (NS), positive control group (C48/80) (Xie et al., 2019), SHLI group and SMI group, with 24 mice in each group. Through a single tail vein injection with optimal doses: NS group (10 mL/kg), C48/80 group (2.5 mg/kg), SHLI group (1293 mg/kg), and SMI group (18.0 mL/kg), mice in each group were sensitized. To determine the strongest positive time of ARs, 24 mice in each drug group were divided into three subgroups based on the time of blood samples were collected: group-1∼3 represents samples taken at 10, 30, 120 min after administration, respectively. Eyeball whole blood samples were collected from each of the eight mice after 10, 30, 120 min of administration, respectively. Then centrifuged at 3000 rpm for 15 min and then cryopreserved at −20°C, part of which was used for enzyme-linked immunosorbent assay (ELISA), and the rest was reserved for subsequent metabolomics research.

ELISA was used in model verification. Histamine and tryptase in mouse plasma were measured according to the methods of their respective ELISA Kit instructions, and the optical density (OD) of each well was measured at 450 nm with an enzyme marker meter. Then, ARs intensity of different drugs and different times were evaluated through the concentrations of histamine and tryptase in plasma samples. Subsequent metabolomics will be based on the optimal ARs model of each injectable drug.

### 2.7 Sample preparation and LC-MS analysis

80 μL of each plasma sample was added to 4 x chromatographic methanol, vortexed for 30 s, stood at 4°C for 1 h and then centrifuged at 12000 rpm for 10 min. The supernatant obtained by centrifugation was centrifuged at 12000 rpm for 10 min and 100 μL of the supernatant was added to the injection flask for detection. Meanwhile, 20 μL of each sample was taken as the quality control sample (QC).

Each sample was injected into the UPLC-Q Exactive-HF MS system equipped with electrospray ionization (ESI) ion source operated in both positive and negative-ion modes (Thermo Fisher, United States). Separation was carried out on hypesil gold column C18 (100 × 2.1 mm, 1.9 μm) (Thermo Fisher, GER), column temperature was set at 40°C; ESI + mode, use 0.1% formic acid as mobile phase A and methanol as mobile phase B; ESI- mode, use 5 mmol/L ammonium acetate (pH = 9.0) as mobile phase A and methanol as mobile phase B; Flow rate: 0.2 mL×min^-1^. Gradient elution began at 98%A- 2%B for the first 0–12 min; at 12–14.1 min, it followed by 0%A- 100%B, and 98%A- 2%B for the final 14.1–17.0 min. Mass spectrometry conditions: capillary temperature, 320°C; spray voltage, 3.2 kV; sheath gas velocity, 40 arb; auxiliary gas flow rate, 10 arb; scanning range, *m/z* 100–1500.

### 2.8 Data analysis

Raw data for metabolomics were preprocessed, and the relevant parameters were screened by compound discoverer 3.1 (CD, Thermo Fisher, USA) library search software. Set relevant parameters for peak extraction, and the quantitative results were normalized. Finally, the quantitative results of the metabolites were obtained. Metabolites with coefficient of variation (CV) less than 30% (Wen et al., 2017) in QC samples were retained for subsequent analysis.

### 2.9 Statistical analysis and differential metabolites identification

The extracted data was processed using MetaX. Principal Component Analysis (PCA) and Partial Least Squares Discriminant Analysis (PLS-DA) were carried out to identify potential biomarkers. Statistical significance was determined by *t*-test (Liang et al., 2012). To obtain qualitative results, retrieve the molecular formula of differential metabolites from databases such as HMDB (https://hmdb.ca/), KEGG (www.kegg.jp) and other databases. Next, input the metabolite names in MetaboAnalysis platform (http://www.metaboanlyst.ca/) to analyze metabolic pathway.

## 3 Results

### 3.1 ARs in mice

#### 3.1.1 Mice behavior study

**FIGURE 2:**
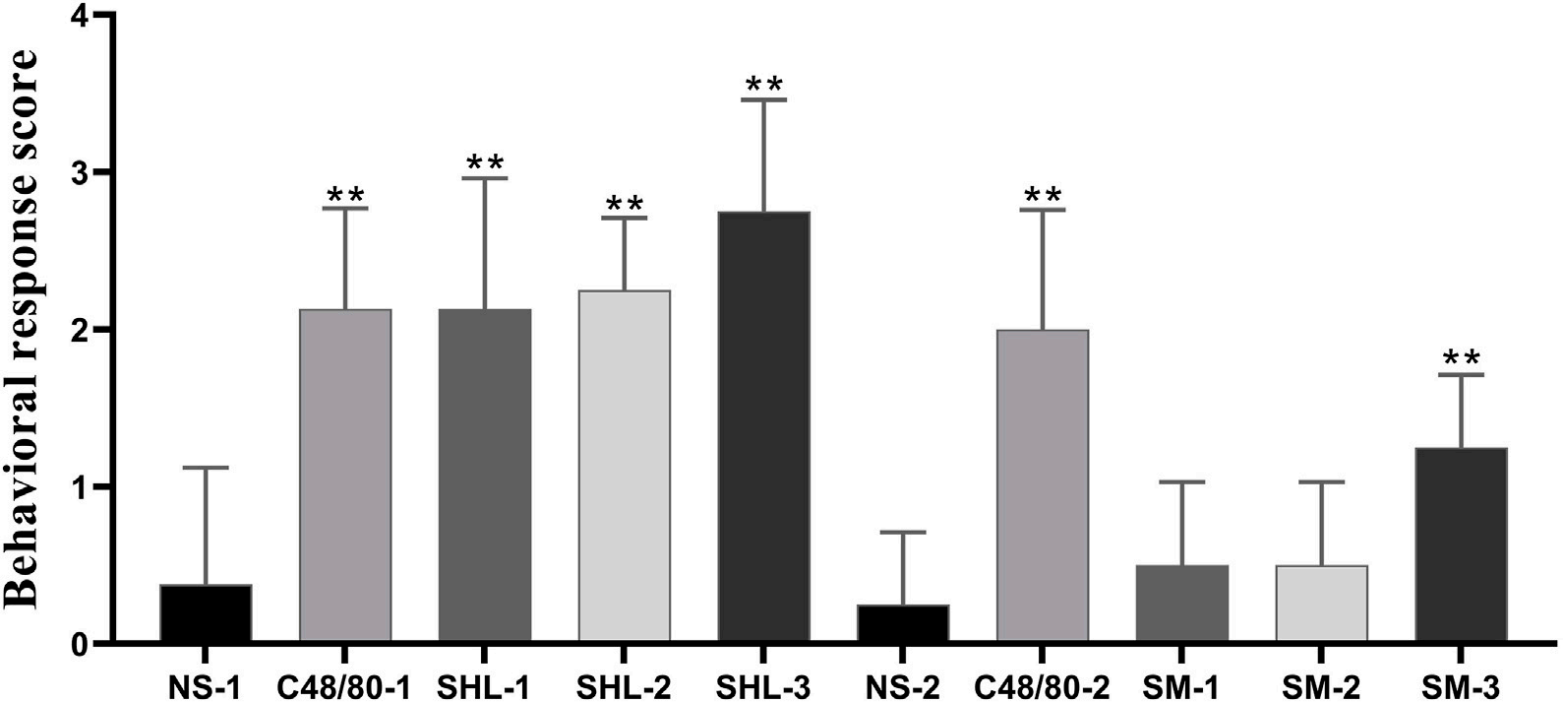
Behavioral response score of each group (SHL and SMI groups were compared with NS-1 group and NS-2 group respectively, *p < 0.05, **p < 0.01).

Firstly, we observed the behavior of mice after administration (Figure 2). The behavioral response level was negative in the NS group and positive in the CV group. There were significant differences between the C48/80–1 and NS-1 groups, as well as between the C48/80-2 and NS-2 groups (*p* < 0.01); The overall response level in the SHLI group was positive, which was significantly different from the NS-1 group (*p* < 0.01), and the behavioral scores increased with the increasing dose in different dose groups. There was no significant change in SM-1 group and SM-2 group compared with NS-2 (*p* > 0.05), while the NS-2 and SM-3 group was significant difference (*p* < 0.01).

#### 3.1.2 Auricle blue staining analysis

**FIGURE 3:**
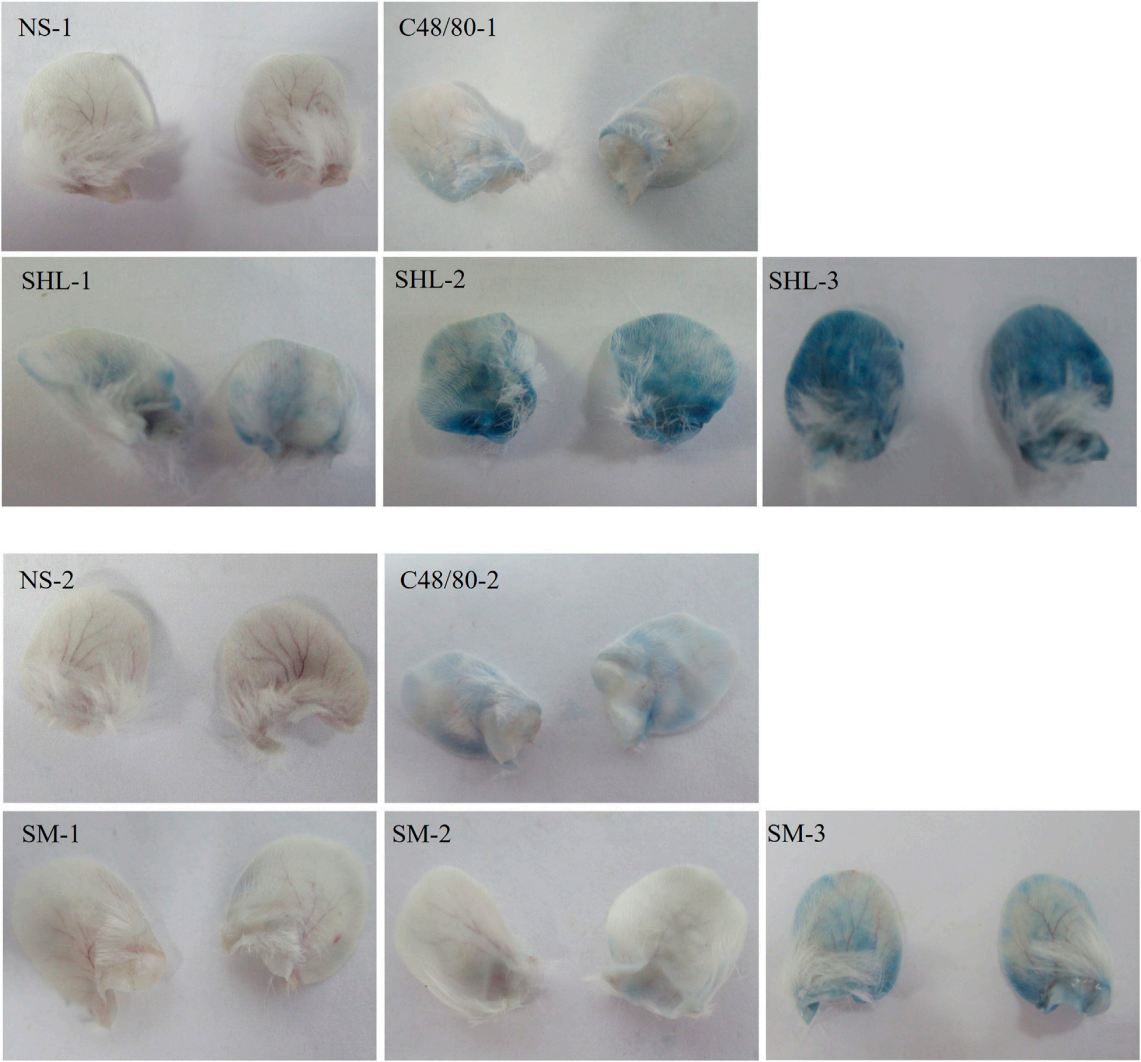
Blue staining of auricles in each group.

An increase in binaural microvascular permeability caused by ARs was detected by auricle blue staining. There was an absence in the NS group, but the degrees of EB blue staining varied among the C48/80 group and each drug treatment group. As the dose increased, the blue-stained area increased, there was a significant difference between SHLI group and NS-1 group (*p* < 0.05). Except SM-1 group, all other SM-injected groups showed significant differences compared to NS-2 group (*p* < 0.05 or *p* < 0.01) (Figure 3).

#### 3.1.3 EB exudation analysis

**FIGURE 4:**
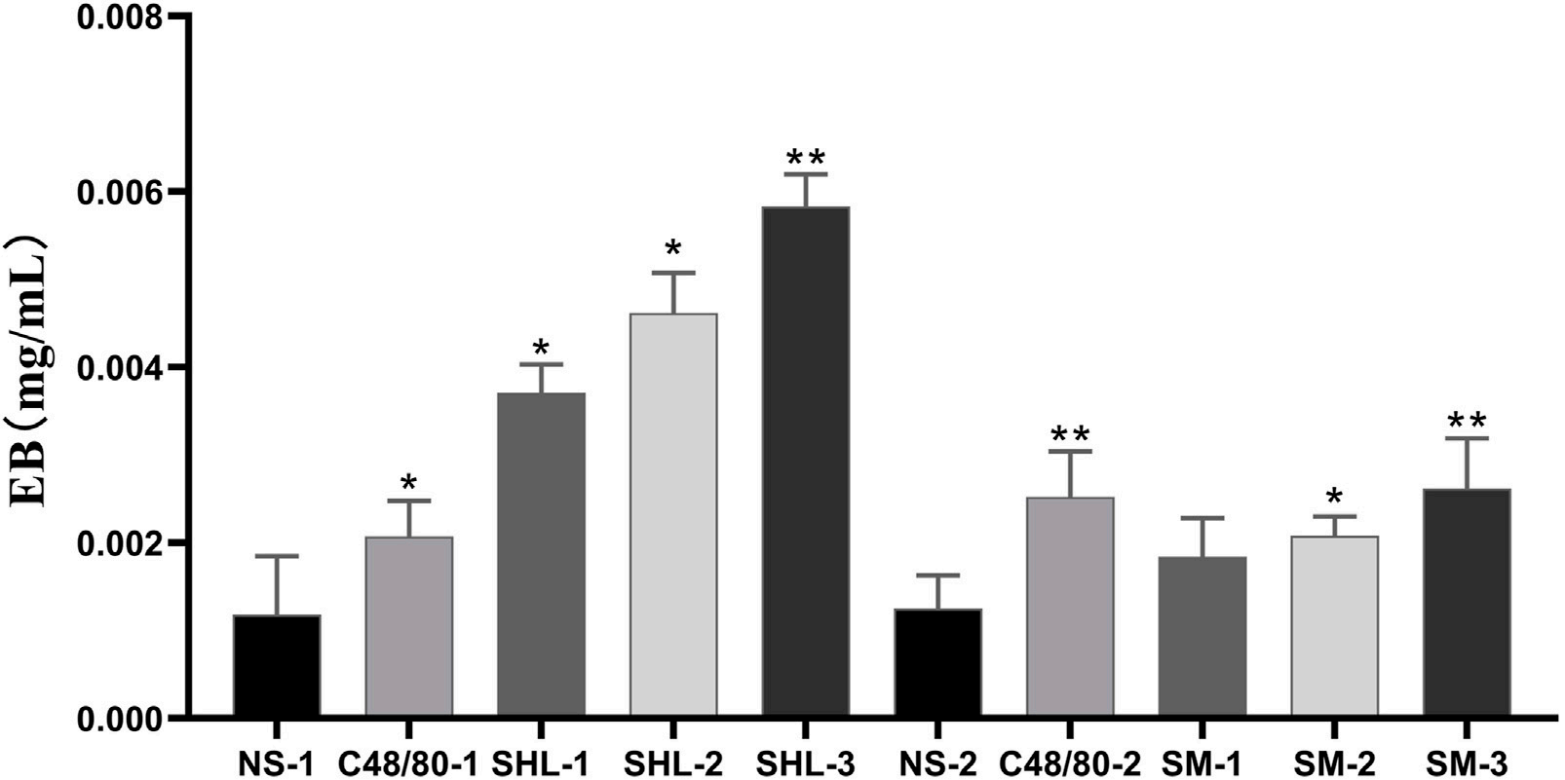
EB Exudation in each group (SHL and SMI groups were compared with NS-1 group and NS-2 group respectively, *p < 0.05, **p < 0.01).

EB standard curve was drawn with the EB concentration as the abscissa and the measured absorbance (A) as the ordinate (y = 66.676x + 0.0088, *R* ^2^ = 0.9991). The degree of EB exudation was calculated. The results indicate that the EB exudation degree in both SHLI group and C48/80-1 group was significantly higher than that in NS-1 group (*p* < 0.05 or *p* < 0.01), and SHLI were higher than C48/80-1. Except SM-1, SM-2 and SM-3 were significantly higher than NS-2 (*p* < 0.05 or *p* < 0.01) (Figure 4).

#### 3.1.4 Dose correlation of ARs

The results showed that the rate of blue staining response, blue staining area score, and the degree of EB exudation increased with increasing dose in both the SHLI and SMI groups, and at the maximum dose, both SHLI and SMI showed the highest sensitivity. The optimal sensitization doses for SHLI and SMI were selected for the follow-up experiment (SHLI groups: 1293 mg×kg^-1^, SMI groups: 18.0 mL×kg^-1^). Meanwhile, we noticed a higher fatality rate in C48/80 without EB and as a result, the dose of C48/80 was reduced in the follow-up experiment.

### 3.2 ARs model verification

**FIGURE 5:**
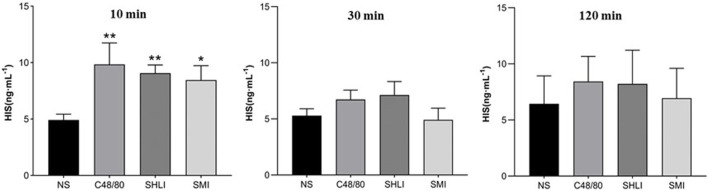
Comparison of histamine concentration between groups in each time period (Compared with NS group, *p < 0.05, **p < 0.01).

**FIGURE 6:**
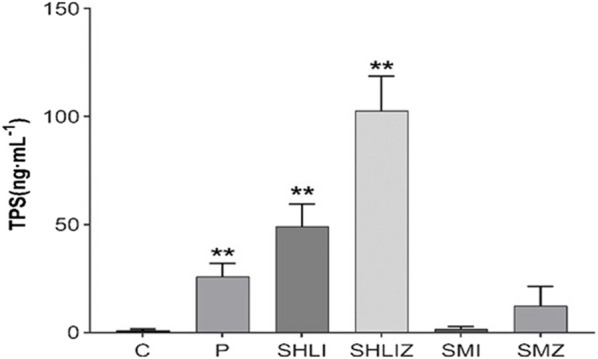
Comparison of tryptase concentration between groups 10 min after administration ( $\bar{x}$ ±s) (Compared with NS group, *p < 0.05, **p < 0.01).

The ELISA results showed that the plasma histamine concentrations at 10 min post dose in the C48/80, SHLI, and SMI groups were significantly higher than those in NS group (*p* < 0.05 or *p* < 0.01), but were not significantly different in each group at 30 min and 120 min post dose from the NS group (Figure 5). Similarly, the plasma tryptase concentrations of C48/80 group and SHLI group 10 min after administration were significantly higher than that of NS group (*p* < 0.05 or *p* < 0.01), but there was no significant difference between SMI group and NS group (*p* > 0.05) (Figure 6). ELISA further verified the AR effect of SHLI and SMI, and the anaphylactoid model was successfully established.

### 3.3 Stability of the LC-MS method

**FIGURE 7:**
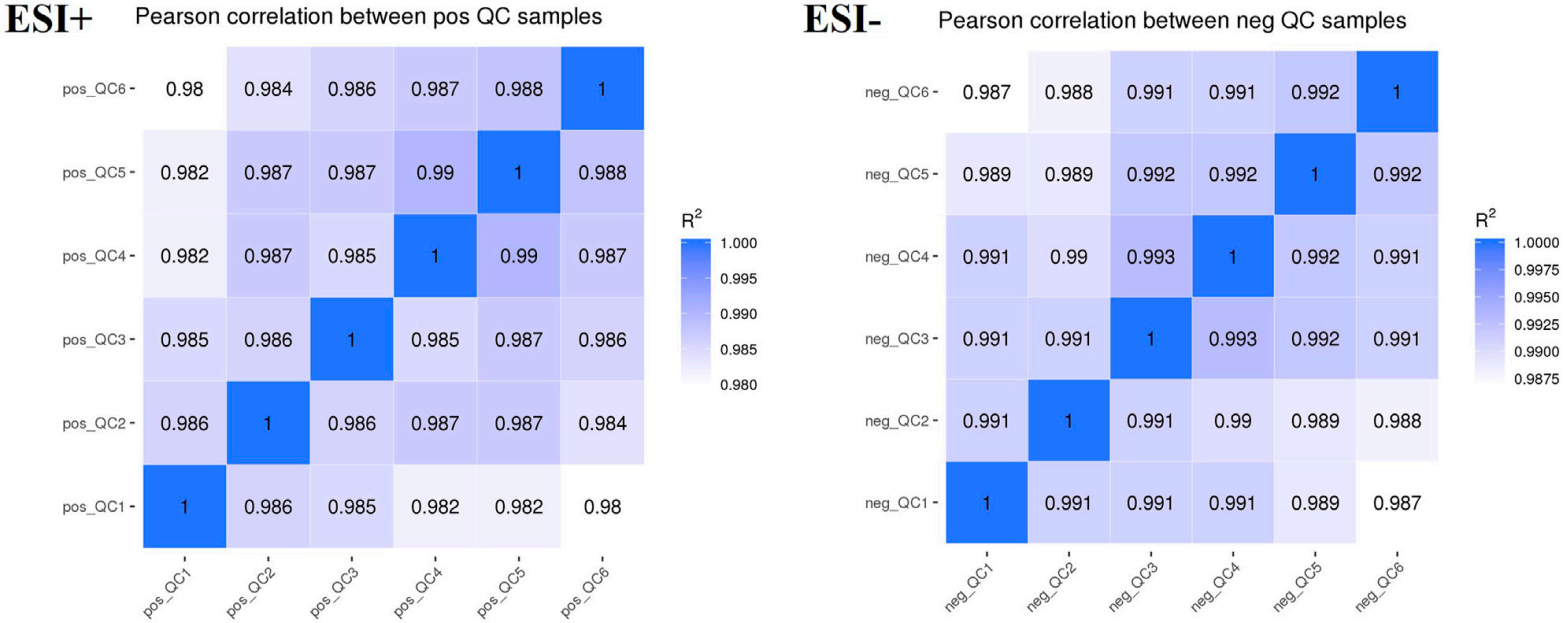
QC sample quality control chart.

Plasma samples were analyzed using an LC-MS system. To monitor the instrument’s detection performance and ensure data stability, QC samples were established and tested throughout the entire process of plasma sample testing. The Pearson’s correlation coefficient (R) between QC samples was calculated based on the relative quantitative value of metabolites. An *R* ^2^ value close to 1 indicates a higher correlation of QC samples. According to the results (Figure 7), the *R* ^2^ value for each QC sample is close to 1, which suggested that the experiment data is stable.

### 3.4 Multivariate statistical analysis for plasma samples

#### 3.4.1 PCA analysis

**FIGURE 8:**
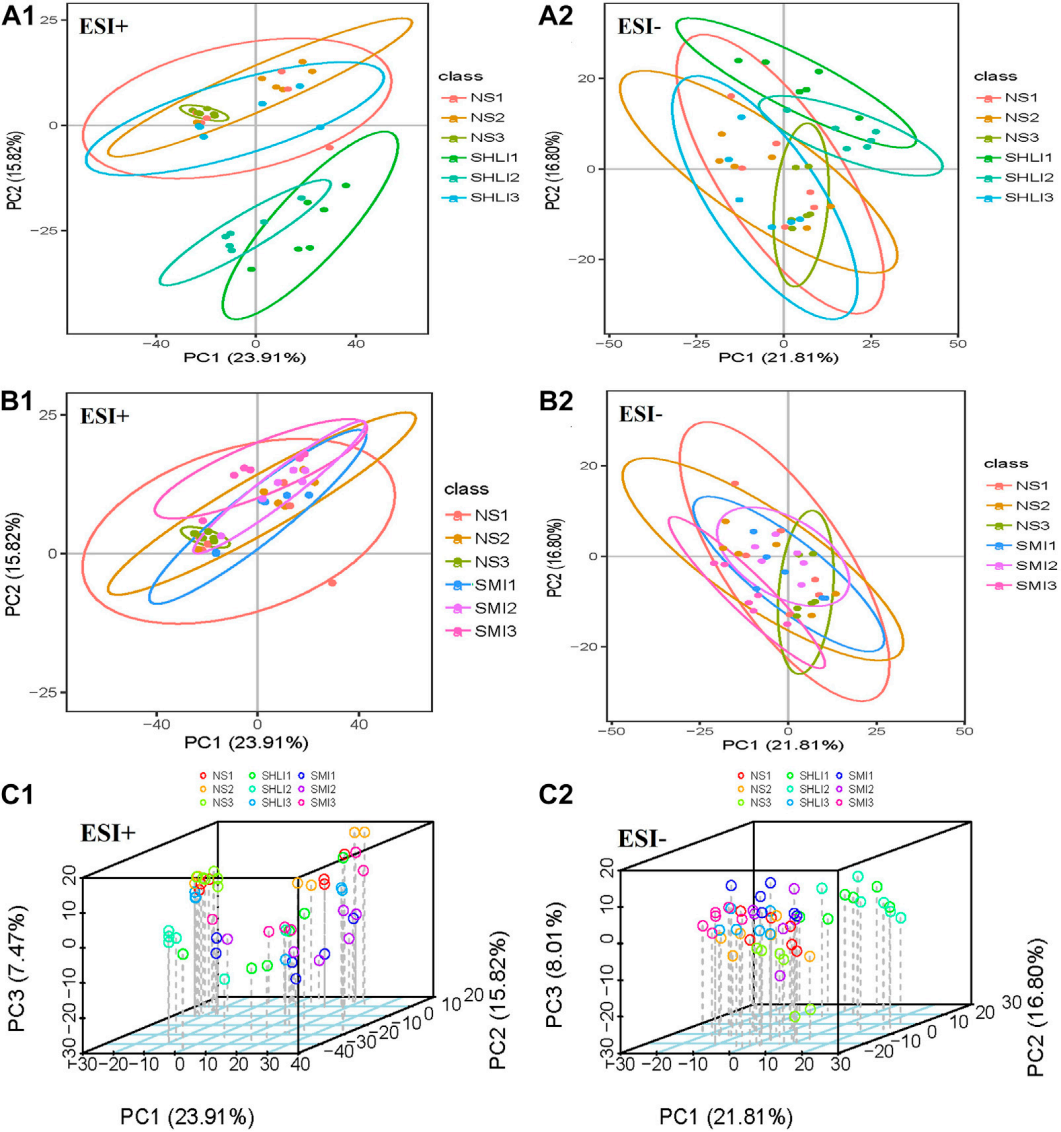
PCA scores plots of mice plasma data **(A1 ∼ A2)**: Comparison of SHLI and NS; **(B1 ∼ B2)**: Comparison of SMI and NS; **(C1 ∼ C2)**: total sample 3D plot.

Unsupervised multivariate principal component analysis (Liu et al., 2020) was used to visually reflect the overall differences between individual group samples. The results were shown in the scatter plot (Figure 8), where the abscissa PC1 and the ordinate PC2 represent the scores of the first and second principal components, respectively. The scatter points of different colors correspond to the samples of different experimental groups. The smaller the difference between the samples, the closer the scattering point will be. It was found that the metabolites of SHLI allergic mice changed greatly in 10 min after dose, while the metabolites tended to return to normal and the AR disappeared in 120 min after dose. This is consistent with the rapid occurrence of ARs. Aggregation occurred between the SMI and NS groups in B1∼B2, indicating that the metabolic difference induced by SMI was relatively small. Based on the previous results, it is considered that the plasma metabolites 10 min after administration could best characterize ARs in mice.

#### 3.4.2 PLS-DA analysis

**FIGURE 9:**
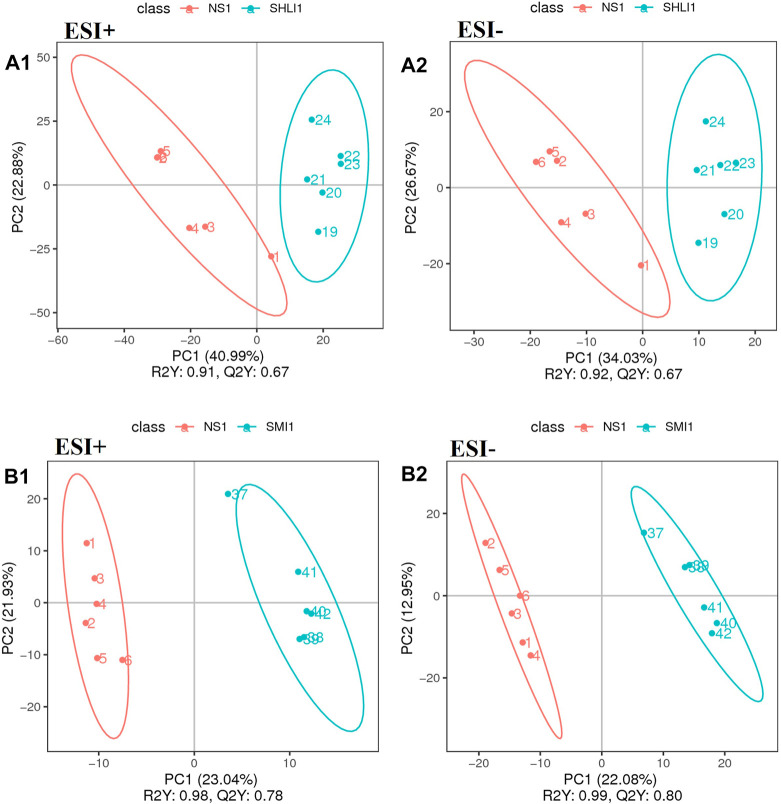
PLS-DA scores plots of mice plasma data **(A1∼A2)**: Comparison of SHLI-1 group and NS-1 group; **(B1∼B2)**: SMI-1 group and NS-1 group.

Multivariate statistical PLS-DA analysis with group supervision was used to find differences in the biochemical composition of the metabolites (Jiang et al., 2019b). According to PLS-DA analysis results of plasma samples taken 10 min after administration, in positive ion mode, *R* ^2^ for SHLI group and SMI group were 0.91 and 0.98, respectively, while their Q^2^ were 0.67 and 0.78, respectively. In negative ion mode, *R* ^2^ for both groups were 0.92 and 0.99, respectively, while their Q^2^ were 0.67 and 0.80 respectively. The results show that the SHLI group, SMI group and NS-1 group can be well separated, and the prediction degree of the model is acceptable (Figure 9).

**FIGURE 10:**
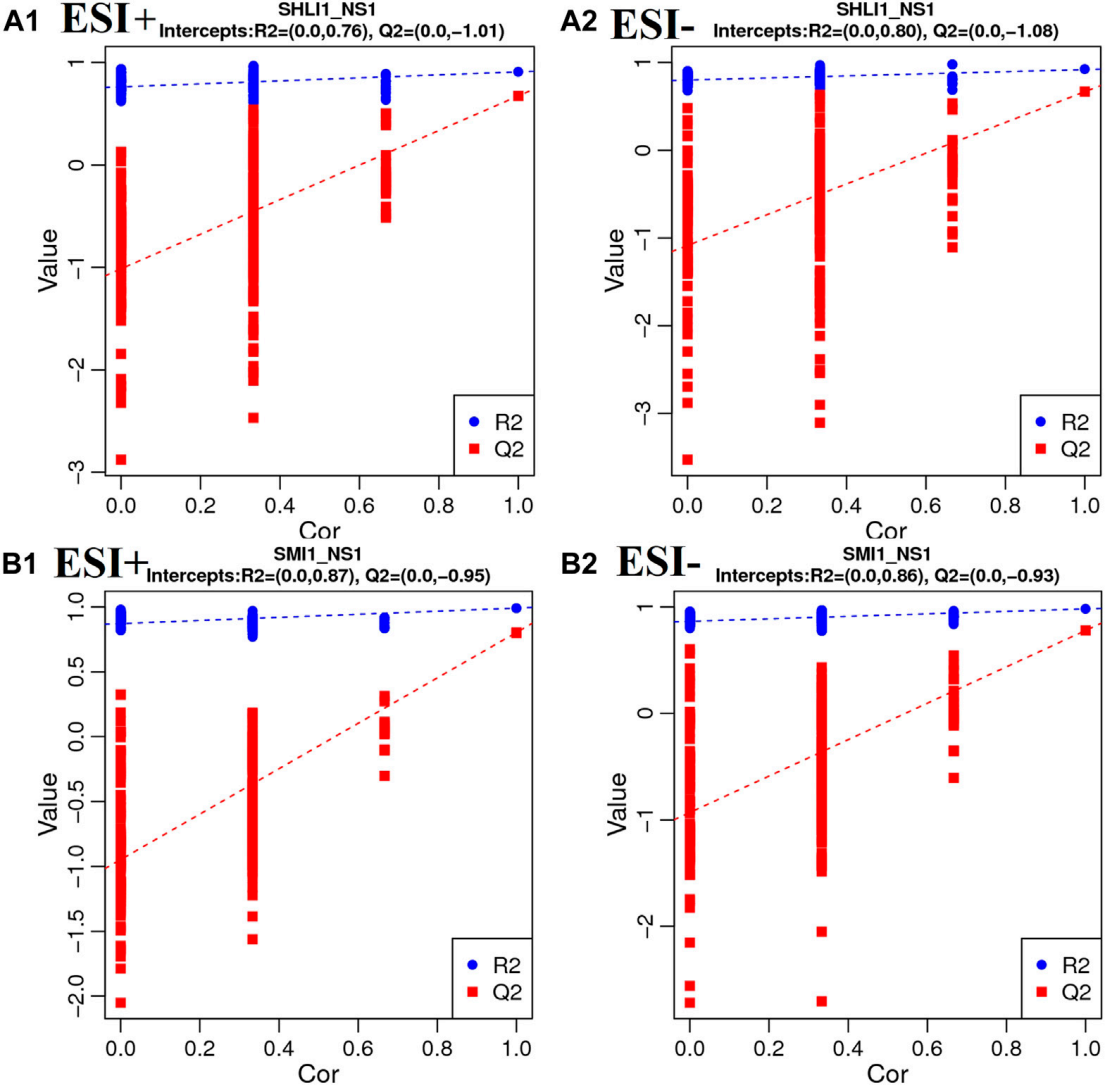
Validation plots with 200 times permutation tests **(A1–A2)**: SHLI1 VS. NS1; **(B1–B2)**: SMI1 VS. NS1.

Moreover, in order to avoid model overfitting, permutation tests were employed to validate the PLS-DA model. Model validation with the number of permutations equaling 200 generated intercepts of *R* ^2^ and Q^2^ of SHLI-1 and SMI-1 in two modes, as shown in Figure 10, intercepts of *R* ^2^ > Q^2^, Q^2^ > 0 in both SHLI-1 and SMI-1 group, suggesting that the established PLS-DA model has prominent fitness and predictability.

#### 3.4.3 Identification of potential biomarkers

Potential biomarkers were identified based on the VIP values and the difference multiple FC values (ratio of the mean value of all biological repeated quantitative values of each metabolite in the comparison group) obtained by PLS-DA model, and the *p*-value of *t*-test. Potential biomarkers which met the criterial (*p* < 0.05, VIP >1, 0.667 < FC > 1.5) should be identified in KEGG and HMDB, and confirmed to participate in the metabolic pathways *in vivo*. Meanwhile, a correlation between metabolites and inflammatory factors was required, factors only involved in anti-inflammatory effects were not taken into consideration. In results, a total of 23 potential biomarkers were identified in SHLI-1 group (Table 3) and 11 potential biomarkers were identified in SMI-1 group (Table 4).

**TABLE 3:** Identification results of potential biomarkers related to SHLI anaphylaxis.

Identification	*m/z*	Formula	Molecular weight	tR (min)	KEGG_ID	HMDB_ID	VIP	Trend	KEGG_pathway
deoxycholic acid	195.13922	C_24_H_40_O_4_	196.14632	13.458	C04483	HMDB0000626	2.2081150	↑	primary bile acid biosynthesis
histamine	112.08714	C_5_H_9_N_3_	111.07998	1.074	C00388	HMDB0000870	2.0811788	↑	histidine metabolism
5-hydroxytryptophan	221.09227	C_11_H_12_N_2_O_3_	220.08472	8.332	C01017	HMDB0000472	2.0581624	↓	tryptophan metabolism
desoxycortone	331.2265	C_21_H_30_O_3_	330.21906	12.504	C03205	HMDB0000016	1.9750519	↑	steroid hormone biosynthesis
N-formylkynurenine	237.08672	C_11_H_12_N_2_O_4_	236.07933	7.313	C02700	HMDB0001200	1.8836979	↑	tryptophan metabolism
17α-hydroxypregnenolone	331.22812	C_21_H_32_O_3_	332.23553	13.601	C05138	HMDB0000363	1.8247771	↓	steroid hormone biosynthesis
progesterone	315.23132	C_21_H_30_O_2_	314.22425	14.082	C00410	HMDB0001830	1.7208383	↓	steroid hormone biosynthesis
prostaglandin H2	351.21774	C_20_H_32_O_5_	352.22497	11.449	C00427	HMDB0001381	1.6485138	↑	arachidonic acid metabolism
cortisone	361.2002	C_21_H_28_O_5_	360.19281	11.623	C00762	HMDB0002802	1.5869097	↑	steroid hormone biosynthesis
nicotinic acid	124.0396	C_6_H_5_NO_2_	123.03236	7.314	C00253	HMDB0001488	1.5768275	↑	nicotinate and nicotinamide metabolism
tryptophan	205.0972	C_11_H_12_N_2_O_2_	204.08994	6.841	C00078	HMDB0000929	1.4910401	↓	tryptophan metabolism
indole	118.06528	C_8_H_7_N	117.05793	7.012	C00463	HMDB0035924	1.4666994	↓	tryptophan metabolism
5-hydroxyindoleacetate	192.07011	C_10_H_9_NO_3_	191.06284	7.355	C05635	HMDB0000763	1.4516224	↑	tryptophan metabolism
hydrocortisone	361.20279	C_21_H_30_O_5_	362.21007	13.195	C00735	HMDB0000063	1.3427945	↓	steroid hormone biosynthesis
estradiol	273.18433	C_18_H_24_O_2_	272.17705	11.407	C00951	HMDB0000151	1.2719107	↑	steroid hormone biosynthesis
3-indoleacetonitrile	175.08664	C_10_H_8_N_2_	174.07944	7.022	C02938	HMDB0006524	1.2658425	↓	tryptophan metabolism
corticosterone	347.2211	C_21_H_30_O_4_	346.21425	11.895	C02140	HMDB0001547	1.2501965	↓	steroid hormone biosynthesis
pregnenolone	317.24689	C_21_H_32_O_2_	316.23978	12.618	C01953	HMDB0000253	1.2342903	↑	steroid hormone biosynthesis
prostaglandin B2	335.22128	C_20_H_30_O_4_	334.21394	11.808	C05954	HMDB0004236	1.1962723	↑	arachidonic acid metabolism
testosterone	289.21616	C_19_H_28_O_2_	288.20869	12.68	C00535	HMDB0002833	1.1899247	↑	steroid hormone biosynthesis
L-kynurenine	209.09183	C_10_H_12_N_2_O_3_	208.08472	5.319	C00328	HMDB0000684	1.1212665	↓	tryptophan metabolism
20-carboxy-leukotriene B4	367.20901	C_20_H_30_O_6_	366.20276	12.652	C05950	HMDB0006059	1.0412087	↑	arachidonic acid metabolism
cholic acid	408.2876	C_24_H_40_O5	407.28	11.968	C00695	HMDB0000619	1.0332030	↑	primary bile acid biosynthesis

**TABLE 4:** Identification results of potential biomarkers related to SMI anaphylaxis.

Identification	*m/z*	Formula	Molecular weight	tR (min)	KEGG_ID	HMDB_ID	VIP	Trend	KEGG_pathway
androsterone	291.23129	C_19_H_30_O_2_	290.22432	15.044	C00523	HMDB0000031	2.4633617	↑	steroid hormone biosynthesis
17α-hydroxypregnenolone	331.22812	C_21_H_32_O_3_	332.23553	13.601	C05138	HMDB0000363	2.4475114	↓	steroid hormone biosynthesis
5-hydroxyindoleacetate	192.07011	C_10_H_9_NO_3_	191.06284	7.355	C05635	HMDB0000763	2.4092249	↑	tryptophan metabolism
progesterone	315.23132	C_21_H_30_O_2_	314.22425	14.082	C00410	HMDB0001830	2.2629566	↓	steroid hormone biosynthesis
serotonin	177.10217	C_10_H_12_N_2_O	176.0949	10.327	C00780	HMDB0000259	2.1652904	↑	tryptophan metabolism
etiocholanolone	273.22113	C_19_H_30_O_2_	272.21361	12.793	C04373	HMDB0000490	2.0307453	↑	steroid hormone biosynthesis
prostaglandin B2	335.22128	C_20_H_30_O_4_	334.21394	11.808	C05954	HMDB0004236	1.9214541	↓	arachidonic acid metabolism
prostaglandin F2α	353.2338	C_20_H_34_O_5_	354.24094	13.175	C00639	HMDB0010199	1.8927133	↓	arachidonic acid metabolism
nicotinic acid	124.0396	C_6_H_5_NO_2_	123.03236	7.314	C00253	HMDB0001488	1.5042402	↓	nicotinate and nicotinamide metabolism
lipoxin B4	351.21844	C_20_H_32_O_5_	352.2256	11.645	C06315	HMDB0005082	1.1939183	↓	arachidonic acid metabolism
prostaglandin H2	351.21774	C_20_H_32_O_5_	352.22497	11.449	C00427	HMDB0001381	1.0198146	↓	arachidonic acid metabolism

**FIGURE 11:**
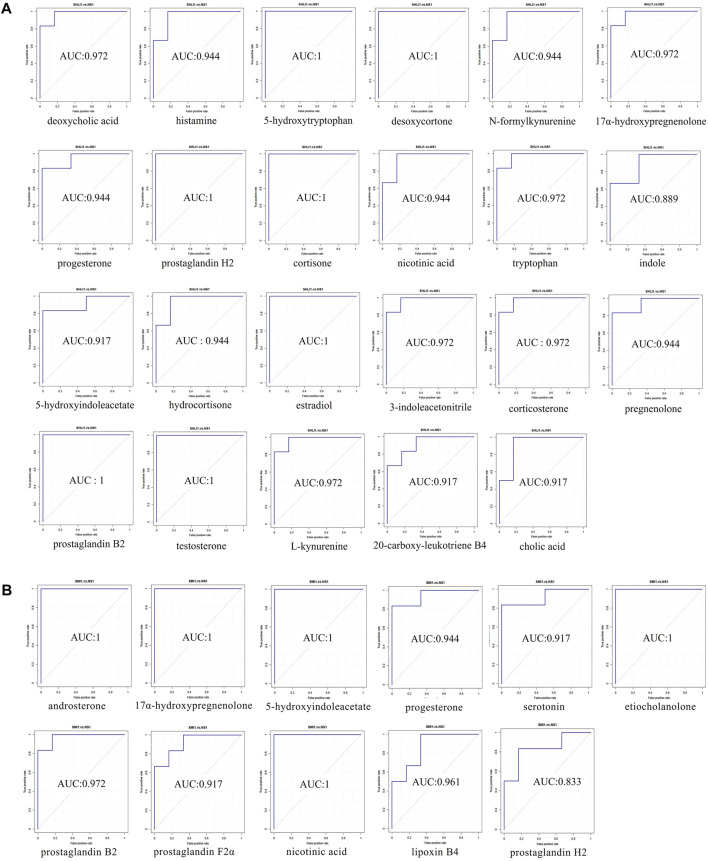
ROC curve plots of biomarkers (**(A)**: biomarkers in SHLI group; **(B)** biomarkers in SMI group).

Receiver operating characteristic curve (ROC) was utilized to evaluate the sensitivity and specificity of biomarkers. With the true positive rate (sensitivity) as the vertical coordinate and the false positive rate (1-specificity) as the horizontal coordinate, the ROC curve was plotted and the area under the ROC curve (AUC) was calculated (Figure 11). The AUC values of biomarkers in both SHLI and SMI groups were close to 1, indicating a high level of predictive accuracy.

### 3.5 Pathway analysis

**FIGURE 12:**
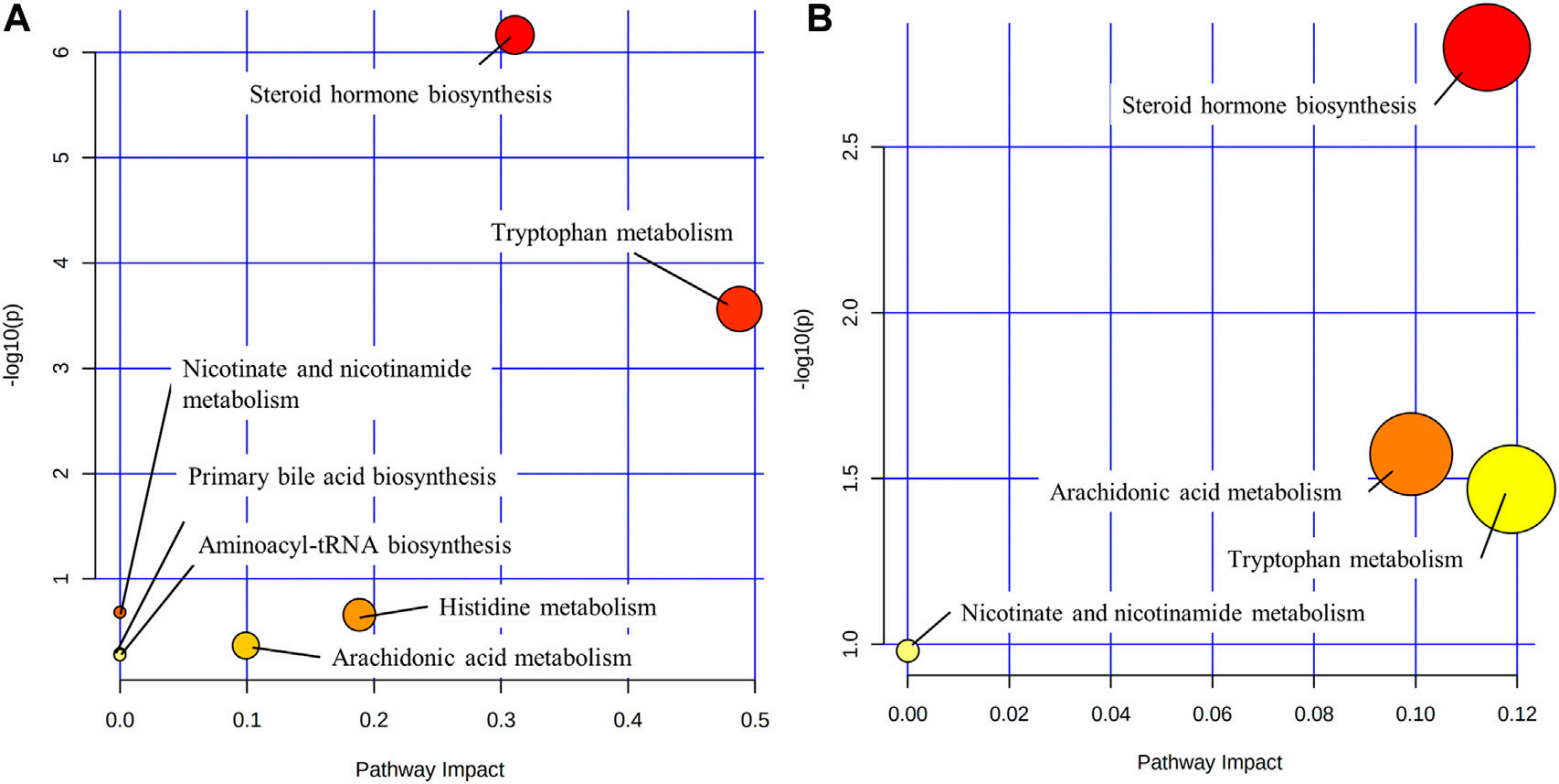
Metabolic pathways (**(A)**: SHLI-1 group related pathways; **(B)** SMI-1 group related pathways).

Metabolic pathways were identified by the MetaboAnalysis platform (http://www.metaboanlyst.ca/). The sequence of associated metabolic pathways in SHLI-1 group were steroid hormone biosynthesis, tryptophan metabolism, histidine metabolism, arachidonic acid metabolism, nicotinate and nicotinamide metabolism, primary bile acid biosynthesis, aminoacyl t (RNA) biosynthesis. The sequence of associated metabolic pathways in SMI-1 group were steroid hormone biosynthesis, arachidonic acid metabolism, tryptophan metabolism, nicotinate and nicotinamide metabolism. The metabolic pathway map of SHLI and SMI was constructed based on the results (Figure 12). The higher *p*-value and Pathway Impact in the figure represents a higher correlation between drug effects and metabolic pathways, that is, more serious disturbances in metabolic pathways.

## 4 Discussion

There are several possible factors that could contribute to TCMI-induced ARs, including variations in bodies and injection types. Different classes of TCMIs are considered to be one of the main factors influencing this phenomenon (Yi et al., 2021). Additionally, quality control issues, such as contamination by exogenous impurities, and the presence of pharmaceutical excipients could also play a significant role. In this study, we focused primally on comparing the anaphylactoid characteristics of two kinds of TCMI with high incidence of ARs—SHLI and SMI. We investigated the relationship between the dose of drugs and hypersensitivity by vascular permeability test and systemic active hypersensitivity test. Our findings show that ARs of SHLI and SMI are dose-dependent, and the sensitization of SHLI is stronger than SMI. At the same dose, the results of vascular permeability test and systemic active hypersensitivity test were in good agreement, indicating the successful research method. In addition, we found the behavioral response of the mice in the SM-2 group was negative, while the auricle blue staining score and EB exudation test showed that the ARs in the SM-2 group was weakly positive. The deviation may be due to the imperfection of the ethological standard for ARs. Therefore, new ethological standard for ARs need to be established.

After investigating the dose-response relationship of ARs induced by SHLI and SMI, we identified several biomarkers in the plasma of SHLI group and SMI group, revealing partially similar metabolic pathways from these potential biomarkers with slight differences. The multiple metabolic pathways identified support the multi-target and multi-pathway characteristics of SHLI and SMI. The different intensity of ARs induced by SHLI and SMI may be related to the partial differences in their metabolic pathways. Moreover, the present known mechanisms of TCMI-induced ARs (Figure 1) include the activation of the RhoA/ROCK signaling pathway, which leads to heightened vascular permeability (Han et al., 2018), activation of the complement system (Galli et al., 2020), and stimulation of G protein-coupled receptors on mast cells (MC), which directly stimulates mast cell degranulation (Yuan et al., 2021). There are potential connections between metabolic pathways and known mechanisms of ARs. The follow-up further analysis of metabolic pathways will help to understand the mechanisms of ARs induced by SHLI and SMI and distinguish between them.

### 4.1 Steroid hormone biosynthesis

Pregnenolone, cortisone, testosterone, estradiol, and desoxycortone in SHLI-1 group were significantly upregulated compared with NS-1 group (*p* < 0.05); Hydrocortisone, corticosterone, 17α-hydroxypregnenolone and progesterone were significantly downregulated (*p* < 0.05). Urinary testosterone and androsterone in SMI-1 group were significantly upregulated (*p* < 0.05), and 17α-hydroxypregnenolone and progesterone were significantly downregulated (*p* < 0.05). Most of these hormone substances are a series of products obtained from cholesterol through enzymatic catalysis, hydroxylation, and redox reactions under the steroid hormone metabolism pathway (Schiffer et al., 2019). Therefore, the significant disturbance of these metabolites may be due to the abnormal activation steroid hormone metabolism pathway. It is speculated to be two situations: As an end product of steroid metabolism and an anti-inflammatory factor (Burke et al., 2015), hydrocortisone was downregulated, while front-end metabolites were upregulated, indicating the inhibition of the hydrocortisone synthesis by administration; Meanwhile, drugs may directly cause the abnormal increase in the synthesis efficiency of testosterone, progesterone and other sex hormones in this pathway. These sex hormones cause pathological changes through lipid metabolism and coagulation function (Langfort et al., 2010), leading to ARs. Hydrocortisone inhibits the activation of complement system (Gewurz et al., 1965). Some sex hormones and gonadal organs can also affect the complement system (Caren and Rosenberg, 1966). The metabolites changing trends of SHLI and SMI groups in steroid hormone biosynthesis are parallel, so it is believed that ARs of the two are related to the abnormal metabolism of steroid hormones. SHLI and SMI may activate the complement system by disrupting steroid biosynthesis and metabolism, leading to further anaphylaxis. The specific mechanism remains to be verified.

### 4.2 Tryptophan metabolism

Serotonin is the central compound of tryptophan metabolic pathway and an inflammatory factor which can stimulate vascular smooth muscle and contract blood vessels in peripheral tissues. Serotonin is also a significant component of mast cell degranulation, and its increased release activates the RhoA/ROCK signal pathway, which further increases vascular permeability and inflammatory exudation (Han et al., 2018; Tao et al., 2020). Tryptophan metabolism is associated with the serotonin pathway, which is highly related to ARs (Diniz et al., 2015). Tryptophan is catalyzed by tryptophan hydroxylase to form 5-hydroxytryptophan, then converted to serotonin under the action of tryptophan decarboxylase and released through synaptic transmission, causing anaphylaxis, serotonin was ended eliminated by enzyme catalysis. L-kynurenine, 3-indoleacetonitrile, 5-hydroxytryptophan, tryptophan and indole in SHLI-1 group were significantly lower, while N-formylkynurenine and 5-hydroxyindoleacetate were significantly higher. It indicates that SHLI participates in the metabolic pathway of tryptophan and promotes the synthesis and release of serotonin. Serotonin and 5-hydroxyindoleacetate in SMI-1 group were significantly higher. Proving that SMI induced ARs by promoting the synthesis and release of serotonin through tryptophan metabolic pathway. SHLI and SMI may promote serotonin release by directly stimulating G protein-coupled receptors on mast cells, leading to MC degranulation. However, further studies are needed to determine their specific mechanism for promoting serotonin synthesis.

### 4.3 Lipid-arachidonic acid metabolism

Significant upregulation of 20-carboxy-leukotriene B4, prostaglandin H2, and prostaglandin B2 occurred in the SHLI-1 group (*p* < 0.05). In contrast, prostaglandin H2, prostaglandin F2α, lipoxin B4, and prostaglandin B2 were significantly downregulated (*p* < 0.05) within the SMI-1 group. The above differential metabolites are all metabolized by arachidonic acid. 20-carboxyl-leukotriene B4 is one of the terminal metabolites of arachidonic acid lipoxygenase pathway and leukotriene is a kind of chemical medium with strong physiological activity, which can improve capillary permeability and strongly stimulate bronchial mucosa, causing respiratory tract inflammation and immune diseases (Di Gennaro and Haeggström, 2014). Arachidonic acid can also be converted to prostaglandin H2 by cyclooxygenase, which is further derived to prostaglandin A, B, E, F, I. Prostaglandins can be intercellular inflammatory mediators to regulate the differentiation of immune cells or the expression of cytokines to promote inflammation (Jang et al., 2020). Among them, prostaglandin F2α is an important pro-inflammatory substance, which can stimulate uterus and vascular smooth muscle (Yao and Narumiya, 2019; Li et al., 2021). Thromboxane is also a derived metabolite of prostaglandin H2 with procoagulant and vasoconstrictive effects (Yuhki et al., 2011; Capra et al., 2014). Lipoxins are arachidonic acid-like substances with anti-inflammatory effects (Chandrasekharan and Sharma-Walia, 2015).

Arachidonic acid metabolism is associated with several known mechanisms of anaphylaxis. The activation of the arachidonic acid metabolic pathway can result in the substantial accumulation of arachidonic acid metabolites. TCMI may prompt MC degranulation by activating G protein-coupled receptors, which can also trigger the activation of Syk kinase and prompt MC to release arachidonic acid metabolites (Shefler and Sagi Eisenberg, 2001). The extravagant release of prostaglandins and leukotrienes leads to inflammation and exudation, which can activate the RhoA/ROCK signaling pathway. This can impact the vascular endothelial cytoskeleton, increase vascular permeability, and exacerbate exudative inflammation (Han et al., 2018).

This indicates that SHLI-induced ARs may be associated with intervention in the arachidonic acid metabolism and promoting the release of leukotrienes and prostaglandins. While SMI caused an overall downregulation of these metabolites, suggesting an anti-inflammatory effect, and that there is no significant correlation between SMI-induced ARs and arachidonic acid metabolic pathway.

### 4.4 Histidine metabolism

Histamine, produced by decarboxylation of histidine under enzymes, is the primary inflammatory mediator of ARs and mainly present in MC. Histamine is also the primary inflammatory substance released by MC degranulation, which is mediated by the activation of the complement system and G protein-coupled receptors. TCMI mainly stimulate the release of pre-synthesized histamine from MC (Jiang et al., 2019a), and strongly stimulate vasodilation. Histamine was significantly upregulated in the SHLI-1 group compared with the NS-1 group (*p* < 0.05), indicating that SHLI stimulate the release of histamine, or interfere with histamine synthesis by histidine metabolism in MC, resulting in ARs.

### 4.5 Nicotinic acid synthesis and metabolism

Nicotinic acid, one of the B vitamins, can be obtained by tryptophan conversion (Davidson et al., 2022), while nicotinic acid deficiency will cause inflammation of the skin and tongue (Ikenouchi-Sugita and Sugita, 2015). Nicotinic acid can also cause ADRs such as skin flushing and itching in the treatment of cardiovascular diseases, which may be due to the vasodilating effect (Kei and Elisaf, 2012). Nicotinic acid was significantly upregulated (*p* < 0.05) in the SHLI-1 group, while it was significantly downregulated (*p* < 0.05) in the SMI-1 group, both of which affected tryptophan metabolism in the body, suggesting that SHLI, SMI may affect the nicotinic acid synthesis and metabolism by perturbing the tryptophan metabolic pathway and leading to ARs.

### 4.6 Primary bile acid biosynthesis

Bile acids are major solutes in bile and act as signaling molecules to activate nuclear hormone receptors to regulate signal transduction. Bile acids are closely related to fat metabolism, glucose metabolism and inflammation regulation of the liver. Studies have shown that the synthesis of bile acids can promote the inflammatory response (Allen et al., 2011). Increased bile acid synthesis can also activate the P38/MAPK pathway (Grambihler et al., 2003), which can stimulate mast cells to release inflammatory mediators (Seo et al., 2009). Bile acids secretion can be regulated by serotonin (Yanchuk et al., 2015). In this experiment, serotonin, deoxycholic acid, bile acid and other bile acids in SHLI-1 group were significantly higher than those in NS-1 group (*p* < 0.05), suggesting that SHLI promotes bile secretion by activating the serotonin release; meanwhile, deoxycholic acid (Xu et al., 2021) can also induce bile acid synthesis and contribute to inflammation, it is speculated that SHLI can cause abnormal metabolism of bile acids, trigger inflammation and lead to ARs.

## 5 Conclusion

In this study, we explored the potential ARs induced by two kinds of widely used TCMI, a LC-MS-based metabolomics was employed to evaluate the mechanisms of ARs. According to the results, high doses of both SHLI and SMI can cause severe ARs, the intensity is positively correlated with the dose. Meanwhile, SMI caused a weaker AR than SHLI, lower dose of SMI hardly induces ARs. Besides, we found a variety of metabolites related to ARs and speculated several metabolic pathways. It was found that SHLI and SMI may promote the simultaneous release of hormones and inflammatory factors by disturbing relevant metabolic pathways, while SMI may also inhibit the release of inflammatory factors in arachidonic acid metabolic pathway, indicating both pro-inflammatory and anti-inflammatory effects. The mild degree of SMI-induced ARs may be related to this. Different types of TCMI may induce ARs by interfering with the similar metabolic pathways, and due to the little difference in the metabolites, the ARs intensity will be different. However, there are several limitations to this study. First, potential effects of excipients and macromolecular impurities present in injections have not been included. Second, the body of clinical disease states were not simulated. This research is still in stages and needs to be further broadened and deepened. In combination with advancement in studying mechanism correlates of TCMI-induced ARs, clarifying the pathological mechanisms and preconized treatment will be the target of further research.

## Data Availability

The original contributions presented in the study are included in the article/Supplementary Material, further inquiries can be directed to the corresponding authors.
