# Supplementary material for: Anaphylactoid reactions induced by Shuanghuanglian injection and Shenmai injection and metabolomics analysis
Source: Front Pharmacol. 2023 Jul 6;14:1200199. doi: 10.3389/fphar.2023.1200199 (PMC10358984; doi:10.3389/fphar.2023.1200199)
Supplement: Supplementary file 1 [file DataSheet1.PDF]

Investigation of the anaphylactoid reaction induced by SHL injection and SM injection and its potential mechanism.

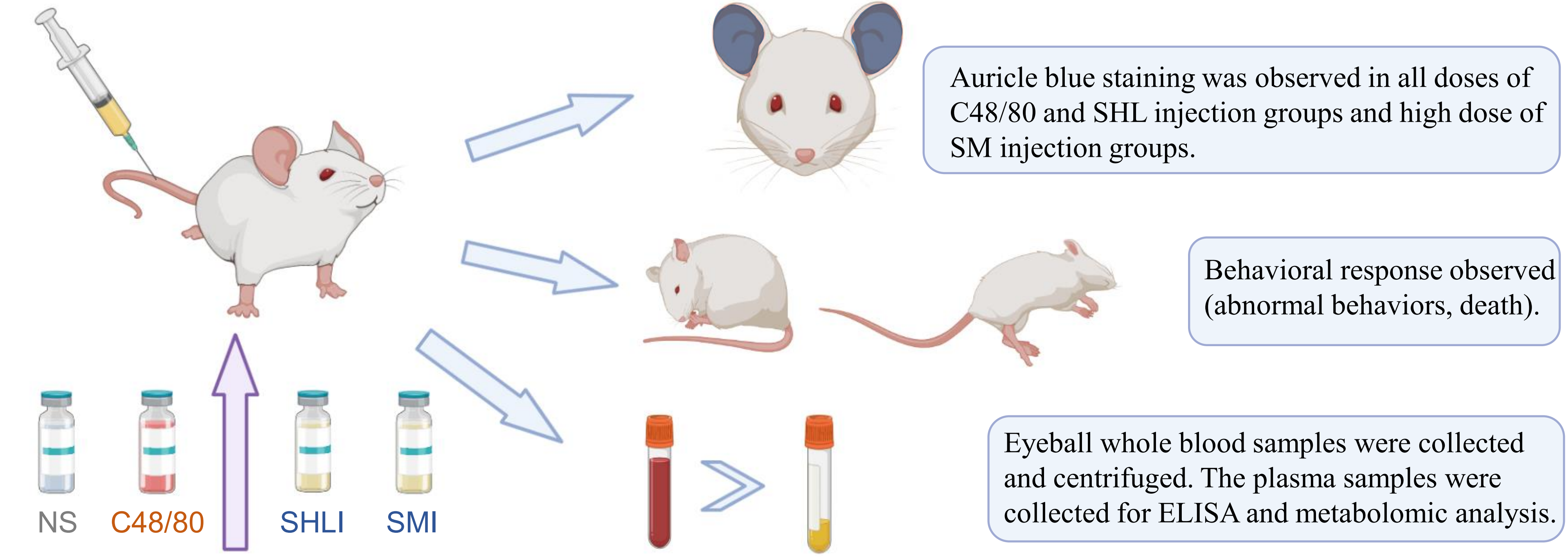

Methods：
ICR mice were treated with normal saline, C48/80, SHL injection and SM injection （containing 0.4% Evans Blue） respectively.

Conclusion:
SHL injection and SM injection may promote the simultaneous release of hormones and inflammatory factors to induce severe anaphylactoid reactions by disturbing steroid hormone biosynthesis, tryptophan metabolism and several relevant metabolic pathways.
